# Knowledge and Attitude Toward Organ Donation Among Interns and Residents in a Tertiary Care Hospital in Gujarat, India

**DOI:** 10.7759/cureus.43797

**Published:** 2023-08-20

**Authors:** Hetvi Tanna, Harshil Patel, Parshva Patel, Gargi Patel, Dinesh Kumar

**Affiliations:** 1 Medical School, Pramukhswami Medical College, Anand, IND; 2 Department of Internal Medicine, Methodist Medical Center, Oak Ridge, USA; 3 Department of Community Medicine, Pramukhswami Medical College, Anand, IND

**Keywords:** transplantation, attitude, knowledge, awareness, healthcare trainees, organ donation

## Abstract

Background

The study aims to assess knowledge and attitudes toward organ donation among medical school interns and postgraduate residents in a tertiary care hospital in Anand, Gujarat, India.

Methodology

A cross-sectional study was conducted among 250 medical school interns and residents of Shree Krishna Hospital, a tertiary care hospital in Anand, Gujarat, India, between March 2021 and March 2022 using a paper questionnaire comprising questions regarding attitudes and beliefs toward organ donation.

Results

Among the 250 participants in this study, 124 (49.6%) were residents, and 126 (50.4%) were interns, with a mean age of 24.18 ± 2.02 years. Of all participants, 88.8% were willing to donate their organs; the main reason was to help people in need. However, the main reason for the refusal to donate organs was the fear of organs being misused/trafficked. Another finding was that 77.2% of the participants had no issue regarding who receives their organs. Only 25.2% of participants had correct knowledge and were aware of the Transplantation of Human Organs Act, 1994 of India, and 66% felt that the current curriculum does not provide sufficient learning experience related to organ donation.

Conclusions

There was less awareness regarding organ donation, despite the willingness to donate organs. Thus, it is essential to increase awareness through curriculum and various workshops to make the process of pledging organs more accessible among those willing to donate. This will play a significant role in addressing the problem and, in turn, help those in need.

## Introduction

Organ donation means the grafting of any human organ from any living person or brain-dead person to some other living person for therapeutic purposes [1]. There is a rise in the number of chronic, devastating diseases of the kidney, heart, lung, and pancreas these days, and often there is a failure of multiple organs, even in younger patients. Organ transplantation can be a life-saving treatment for these patients [2].

Healthcare workers are in direct contact with the general population, as well as patients suffering from end-organ diseases and their families, on a daily basis. Thus, they very well know about the condition of such patients and the importance of organ donation. They can also educate and spread awareness about the importance and need for organ donation to the general population. Therefore, assessing the knowledge and attitude of healthcare workers toward organ donation is necessary.

Most patients on the organ transplant waiting list never get a chance to receive one. There is a massive gap between the demand and supply of human organs for transplantation [3]. As of 2021, the organ donation rate in India stands at 0.4 per million population [4]. In India, there is a need for about 500,000 organs each year. Unfortunately, only 2-3% of the demand is fulfilled, resulting in numerous deaths due to organ failure annually across the country [5]. In 2022, India underwent 11,423 kidney transplants, 3,718 liver transplants, and 250 heart transplants [6]. In India, approximately 1,50,000 brain deaths occur each year due to accidents. If 5-10% of these deaths result in successful organ donation, there may be no need for living individuals to donate organs. Thus, raising awareness is crucial and must be done at all levels, including community, public/patient, hospital, and government-initiated programs [7].

A medical school intern (also described as an intern in this study) is a physician in training who has completed the medical school curriculum but does not have an unrestricted license yet to practice medicine unsupervised. Interns work under the supervision of fully licensed physicians in a teaching hospital setting for one year as a part of medical school requirements in many countries. A postgraduate resident (also described as a resident in this study) is a medical school graduate and doctor in training taking part in a graduate medical education program in a specific specialty for three to seven years after finishing medical school. These various specialties include but are not limited to internal medicine, general surgery, orthopedics, ophthalmology, pathology, psychiatry, anesthesia, and dermatology. We specifically included interns and residents in our study because they represent a link between medical students and practicing physicians as well as the general population in the community. They are still under supervision and undergoing medical training. Thus, increasing their awareness and knowledge about organ donation can have a higher impact on future outcomes.

A study conducted at a medical college in Karnataka, India, found that while most interns and postgraduate residents had sufficient knowledge about organ donation, there were still gaps in their understanding. Interestingly, according to the study, only 3.3% of interns and 14% of postgraduates had registered for organ donation [8]. Providing adequate education and training to healthcare professionals can enhance their willingness to volunteer for organ donation. Moreover, they can assist in promoting awareness of organ donation among their patients, which could ultimately improve the practice of organ donation.

Another study suggested that organ donation should be incorporated in the early stage of the medical curriculum. Most (91.2%) students felt the need to revise the medical curriculum on organ donation [9]. So, the assessment regarding knowledge of organ donation among interns and residents is essential.

## Materials and methods

The study’s primary purpose was to evaluate awareness, attitude, and knowledge of organ donation among interns and residents in a tertiary care teaching hospital. This study was conducted at Shree Krishna Hospital in Anand, Gujarat, India. It was a cross-sectional observational study conducted over a period of one year between March 2021 and March 2022.

Study population

The eligible study populations were all interns and residents at Shree Krishna Hospital between March 2021 and 2022. A random representative sample was taken from interns and residents from different departments of internal medicine, general surgery, pathology, psychiatry, ophthalmology, anesthesia, orthopedics, and dermatology. Participants were approached at their workplace personally. At initial contact, the aim and objectives of the study were explained. Participants were informed that participation was voluntary, and they had the right to withdraw at any time. Standardized verbal consent was taken before the initiation of data collection. They were ensured of the confidentiality of collected information. Out of 325 eligible interns and residents, 298 were contacted. A total of 250 agreed to participate in this study. Out of 48 people who denied participation, 20 were interns, and 28 were residents.

Data collection

Data were collected by a pretested questionnaire which includes questions regarding knowledge and attitude toward organ donation. Questionnaires contained multiple-choice questions except for age. We have attached the questionnaire in the supplemental material. Questionnaires were filled out by participants in front of the researcher. Filled questionnaires were stored in Health Insurance Portability and Accountability Act-compliant storage. Data were manually entered in an Excel sheet by two individual researchers and double-checked by another researcher for validity. No other invasive procedures or investigations were conducted on participants.

Data analysis 

Participants’ characteristics are shown in tabular form. Categorical variables are displayed in percentages. We used the chi-square test to identify the difference between these categorical variables. Continuous variables are displayed in mean ± standard deviation. We used the t-test to identify differences in continuous variables. A p-value of less than or equal to 0.05 was considered statistically significant. Data were stored in Microsoft Excel, and we used the SPSS software version 16.0 (SPSS Inc., Chicago, IL, USA) for data analysis.

Ethical considerations

Ethical approval was obtained from the Institutional Ethics Committee (approval number: IEC/HMPCMCE/2019/Ex. 16/211/2021). Verbal consent was taken. The participants were not asked to write their names on the questionnaire, so their privacy was maintained. The data obtained were used just for the purpose of the study, and confidentiality was maintained.

## Results

Among the 250 participants in this study, 49.6% (n = 124) were postgraduate residents, and 50.4% (n = 126) were interns at a tertiary care hospital, with a mean age of 24.18 ± 2.02 years. Of them, 91.2% were Hindus, 2.4% were Muslims, 2.4% were Jains, 0.4% were Christian, and 3.6% did not reveal their religion. The total number of males in this study was 50.4% (n = 126), and the number of females was 49.6% (n = 124) (Table 1).

**Table 1:** Distribution of study participants by gender, religion, and designation.

Characteristics	Mean ± SD or Frequency (%)
Age in years	24.18 ± 2.02
Gender	
Female	124 (49.6%)
Male	126 (50.4%)
Religion	
Hindu	228 (91.2%)
Muslim	6 (2.4%)
Christian	1 (0.4%)
Jain	6 (2.4%)
Didn’t specify	9 (3.6%)
Designation	
Resident	124 (49.6%)
Intern	126 (50.4%)

Correct knowledge regarding the Organ Donation Act was found to be very low among study participants. Only 30.6% (n = 38) of total residents and 19.8% (n = 25) of interns were aware of the Transplantation of Human Organs Act, 1994 of India. The majority of the participants, 74.8% (n = 187), either did not know the year of the Act or were unaware of any such law (p = 0.492) (Table 2). No significant difference exists between interns’ and residents’ knowledge regarding the Organ Donation Act.

**Table 2:** Correct knowledge regarding the Organ Donation Act 1994. χ^2 ^= 3.8691; df = 1; p-value = 0.492.

Correct knowledge	Residents	Interns	Total
Yes	38 (30.6%)	25 (19.8%)	63 (25.2%)
No	86 (69.35%)	101 (80.1%)	187 (74.8%)
Total	124 (49.6%)	126 (50.4%)	250 (100%)

Of the study participants, 61.2% (n = 153) knew that some organ donation activity was carried out at Shree Krishna Hospital. Among these, only 98 participants knew correctly that only cornea procurement was done at this tertiary-level hospital.

**Figure 1:**
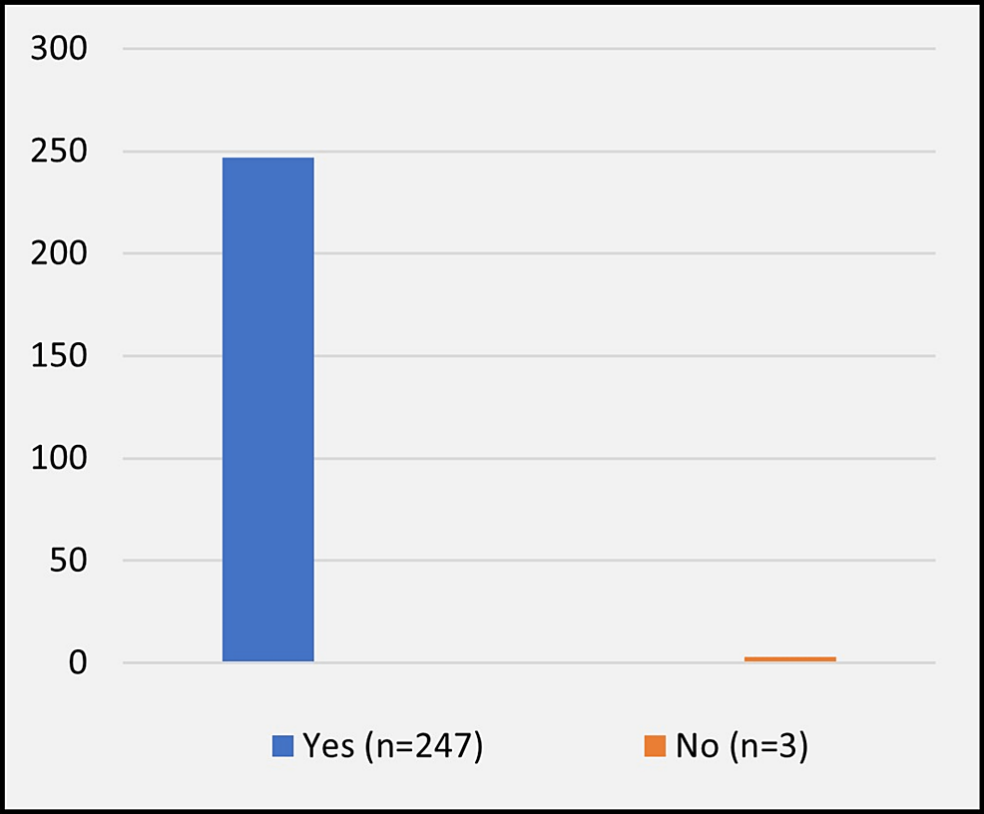
Is organ donation a need or not?

When asked about their views on the necessity of organ donation, 98.8% (n = 247) agreed that it is indeed necessary (Figure 1).

Of all the participants, 22% reported that either one of their family members or friends had donated organs to someone. None of the participants had ever donated any organ. However, 33.6% (n = 84) had donated blood, and one participant had donated bone marrow.

The study participants were asked about their willingness toward organ donation. Most of them, 88.8% (n = 222), were willing to donate their organ. While 11.2% (n = 28) did not wish to donate their organ in the future, of which 46.42% were interns and 53.57% were residents (p = 0.656) (Table 3). It was found that multiple reasons were chosen by participants for their willingness toward organ donation (Table 4). The most common reason for willingness to organ donation was found “to be helpful to the needy” (n = 175), and other reasons were “contribute to the medical science” (n = 40) and “to be useful after death” (n = 63).

**Table 3:** Participants who wish to donate their organs. χ^2^ = 0.1988; df = 1; p-value = 0.656.

Wish to donate	Residents	Interns	Total
Yes	109 (87.9%)	113 (89.6%)	222 (88.8%)
No	15 (12.09%)	13 (10.31%)	28 (11.2%)
Total	124 (49.6%)	126 (50.4%)	250 (100%)

**Table 4:** Reasons for willingness to donate organs among participants (n = 222). Multiple reasons were chosen by a single person.

Reasons	Frequency (%)
Know about the benefit of organ donation to the needy and want to help	175 (78.82%)
Contribute to medical science	40 (18.01%)
Want to be useful after death	63 (28.37%)

According to the participants, the primary reason for their hesitation toward organ donation was the fear of organ misuse or trafficking, with seven individuals expressing this concern. They also feared that the process of harvesting organs would disfigure their body (n = 5). One of the participants also felt that organ donation is against their religious beliefs (Table 5).

**Table 5:** Reasons for refusal to donate among participants (n = 28).

Reasons	Frequency (%)
Afraid the process of harvesting will disfigure the body	5 (17.85%)
Against religious belief	1 (3.5%)
Afraid that organs will be misused/trafficked	7 (25%)
Not specified/ others	15 (53.57%)

Out of those who wanted to donate their organs, only 13 (5.2%) participants had actually pledged to do so and possessed donor cards. Of these 13, only two individuals always carried their donor cards with them. Postgraduates (7.2%) were found to have more donor cards than interns (3.17%). The vast majority of participants, 94.8% (n = 237), did not have a donor card as they had not yet made the decision to pledge their organs (Table 6).

**Table 6:** Have a donor card.

Have a donor card	Residents	Interns	Total
Yes	9 (7.2%)	4 (3.17%)	13 (5.2%)
No	115 (92.7%)	122 (96.8%)	237 (94.8%)
Total	124 (49.6%)	126 (50.4%)	250 (100%)

**Figure 2:**
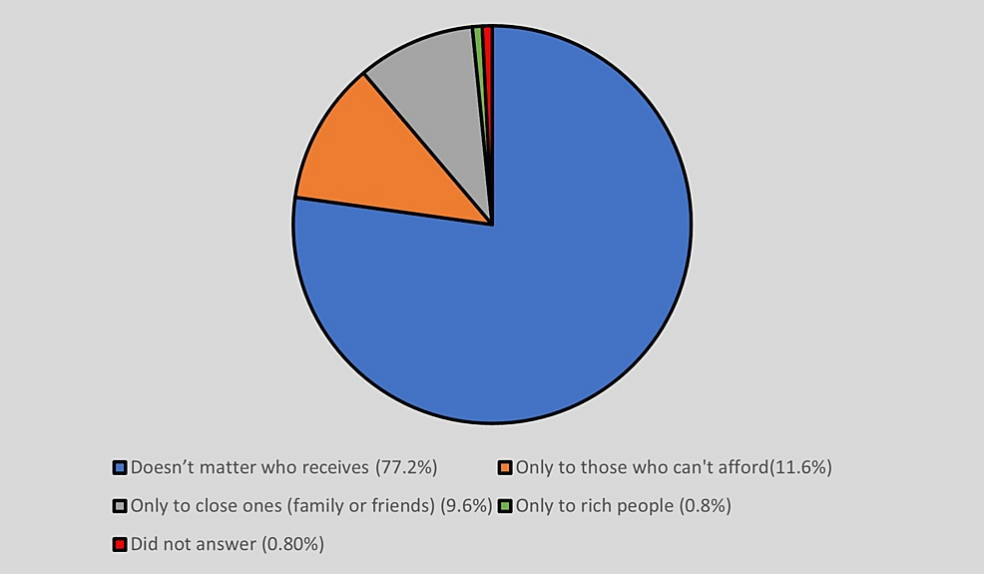
Preference of who can receive their organ.

According to the study, the majority of participants, 77.2% (n = 193), did not have any concerns about who receives their organs. However, some participants had preferences for specific recipients. For example, 11.6% (n = 29) preferred that their organs be given to those who cannot afford them, 9.6% (n = 24) preferred donating only to their close ones like family or friends, and two (0.8%) preferred to donate to wealthy individuals (Figure 2). Two (0.8%) did not answer this question.

From the survey, 67.6% (n = 169) of the participants believed their family would support their decision to donate organs. Also, 27.2% (n = 68) of participants had never discussed this matter with their families. Other 4.8% (n = 12) participants said their family would not support their decision to donate organs. When participants were asked whether they would agree if one of their family members wished to donate organs, 82.8% (n = 207) said they would agree with their decision. However, 4.4% (n = 11) will not agree with the decision of their family member, and 12.4% (n = 31) of participants had never thought about this.

Overall, 11.6% (n = 29) of participants or their family members had benefited from organ donation. Only 12% (n = 30) of the participants had attended any organ donation-related training/sensitization program within the past year, of which 53.3% were interns and 46.6% were residents. The majority of them (88%) had not attended any such organ donation-related program within the last year. The majority of them, 66.4% (n = 166) (85 interns and 81 residents), felt that the current curriculum did not provide sufficient learning experience related to organ donation (Table 7). Thus, there is a need for more such programs to be organized to increase awareness regarding organ donation.

**Table 7:** Is the current curriculum sufficient? χ^2 ^= 0.1278; df = 1; p-value = 0.721.

	Residents	Interns	Total
Yes	43 (34.67%)	41 (32.54%)	84 (33.6%)
No	81 (65.32%)	85 (67.4%)	166 (66.4%)
Total	124 (49.6%)	126 (50.4%)	250 (100%)

## Discussion

The disparity between the number of organs available for donation and the number of patients requiring organs is vast. Interns and residents are the future of healthcare in any country, and their attitudes and practices toward organ donation influence the outcome of those people in need. This study provides insights into the knowledge and attitudes toward organ donation of interns and residents working at Shree Krishna Hospital.

The study was conducted among 250 participants, of whom 124 (49.6%) were residents and 126 (50.4%) were interns. Only 30.6% (n = 38) of total residents and 19.8% (n = 25) of interns were aware of the Transplantation of Human Organs Act, 1994 of India. This reflects a scarcity of awareness regarding the laws and regulations of organ donation.

In our study, 22% of the participants shared that their family member or friend had donated organs to others. A separate study conducted among interns in Telangana found that 10.6% had family members who donated their organs [10].

It was found that 89.6% of interns and 87.9% of residents (88.8% overall) were willing to donate their organs. In a similar study conducted in Telangana, 77.5% of the participants were willing to donate their organs [10]. In a separate study conducted among healthcare professionals in a rural town in the Konkan region of Maharashtra state, the willingness for organ donation was found to be low (56.3%) [11], while in another study conducted among medical undergraduates in PGIMS at Rohtak, 85% of the subjects were willing to donate their organs [12].

The study participants were asked about their willingness toward organ donation. It was found that participants chose multiple reasons for their willingness toward organ donation. The most common reason for willingness to organ donation was found to be helpful to the needy (78.82%), and other reasons were to contribute to medical science (18.01%) and to be useful after death (28.37%). A study from the United States found religion to be a major guiding factor for willingness to donate organs [13].

In our study, 11.2% (n = 28) did not wish to donate their organ in the future, of which 46.42% were interns and 53.57% were residents. Among them, the main reason was that the participants were afraid of their organs being misused/trafficked (25%). They also feared that the process of organ procurement would disfigure their body (17.85%), and 3.5% of the participants felt that organ donation was against their religious beliefs and, thus, were unwilling to donate. In a study conducted in Telangana, the most common reason for not being an organ donor was found to be fear of organ misuse (55.5%), followed by fear of being declared dead earlier if an organ donor card was signed (27.8%) [10]. In another study conducted in Karnataka, reasons for a lack of willingness to donate organs included fear of the organs being wasted (56.1%), misuse of organs (26.9%), or mutilation. In the study conducted in Karnataka, around 14.6% quoted religious beliefs as their reason for not donating organs [8]. In a study conducted in Chennai, religion was not the matter when considering organ donation for 94.54% of the participants [14]. Other studies in Bangalore and rural Puducherry came to similar conclusions regarding religious factors governing decisions regarding organ donation [15,16]. Thus, religious beliefs are the least common reason for objections to organ donation.

In our study, 22% of the participants revealed that someone in their family or friends donated organs to someone. None of the participants had ever donated any organ. However, 33.6% (n = 84) of participants donated blood, and one participant donated bone marrow. In a study conducted in urban Puducherry among the general population, only two out of 100 (2%) participants had donated their blood. This shows positive blood donation practices among medical professionals compared to others [17].

The study also showed that 77.2% of the participants had no issue regarding who receives their organs. But some participants preferred that their organs should be received only by those who cannot afford them (11.6%); some wanted to donate to their close ones like family or friends only (9.6%), and some to rich people (0.8%). In another study conducted in Karnataka, 72.33% agreed they would donate to anyone in need, and 27.67% were willing to donate organs only to family members or friends [8]. This reflects that most participants are open to donating organs to needy people without any preferences.

Among those who wished to donate, only 13 (5.85%) participants had pledged their organs and had donor cards with them. Out of the 222 individuals who expressed a willingness to donate, only two reported carrying their donor cards at all times. It is interesting to observe that a higher percentage of postgraduates (3.6%) than interns (1.6%) had donor cards among all participants. The vast majority, 237 individuals (94.8%), did not possess a donor card because they had not pledged to donate their organs yet. In a study in Kerala, of the 86 participants who were firmly willing to donate, only 14 participants possessed an organ donation card [9]. The number of participants with donor cards was low (5.6%) in a study conducted in Telangana [10]. This shows that the number of people with donor cards among those willing to donate is low. To tackle this issue, a potential solution could be to merge donor cards with driver’s licenses or Aadhar cards (it is a government ID that contains a unique identification number given to Indian citizens), thereby reducing the need to carry multiple cards. This would not only simplify the process of becoming an organ donor but also spread awareness, which could lead to more individuals willing to participate in this life-saving initiative.

The study found that 66% (85 interns and 81 residents) felt that the current curriculum did not provide sufficient learning experience related to organ donation. Two studies in Kerala had similar findings [9,18]. Another study conducted in Bangalore found that first-year medical students had a positive attitude toward organ donation, but the curriculum was insufficient [19]. Thus, adding timely workshops and sessions about organ donation to the curriculum can be quite helpful to increase awareness.

The rates of organ donation in the general population may be affected by cultural and social factors. Certain traditional beliefs, such as the significance of preserving the body’s integrity after death, may cause hesitation when considering organ donation. Family decision-making can also make it challenging for an individual to decide on organ donation, as everyone’s opinion might differ. Additionally, communication may pose a barrier as information about organ donation is not always available in local languages, making it difficult for individuals to comprehend and contemplate the option.

One donor has the potential to save eight lives and improve the quality of life for over 75 others [20]. Organ donation is a crucial act of kindness for those in need. Spreading awareness and encouraging more people to pledge their organs willingly can have a significant impact. Conducting workshops and seminars to train healthcare professionals is an effective way to educate and inspire the general population. This will help increase the organ donation rate, significantly benefit those on transplant waiting lists, and improve their lives.

The strength of this study is that it represents a subgroup of the population in western India with a limited number of previously published studies assessing their knowledge and attitude toward organ donation. We also have included interns and residents who are still in medical training and have exposure to both the clinical world and the teaching side of medical school. Some of the limitations of this study are generalizability to the general population as it was only conducted among interns and residents. This study had a relatively small number of participants compared to a large population-scale size study. Future research is needed to assess the knowledge of all healthcare workers, including ancillary staff.

## Conclusions

The vast deficit in the number of organ donations can be improved by amending the gaps in the knowledge and awareness of the future healthcare workers on whose shoulders rests the burden of educating and motivating the masses for organ donation. This feat must begin with the inclusion of organ donation in the current curriculum in a more thorough manner. Regular reinforcement of this knowledge through seminars, workshops, talks by experts, and reading material is essential. Misbeliefs regarding organ donation among healthcare workers must be carefully sought and removed. The pledging of organs for donation should be made more accessible, and the importance of carrying a donor card should be emphasized. Alternative ways such as merging a donor card with any other government ID like a driving license or Aadhar card can solve the problem of carrying multiple cards.

## Figures and Tables

**Figure 3:**
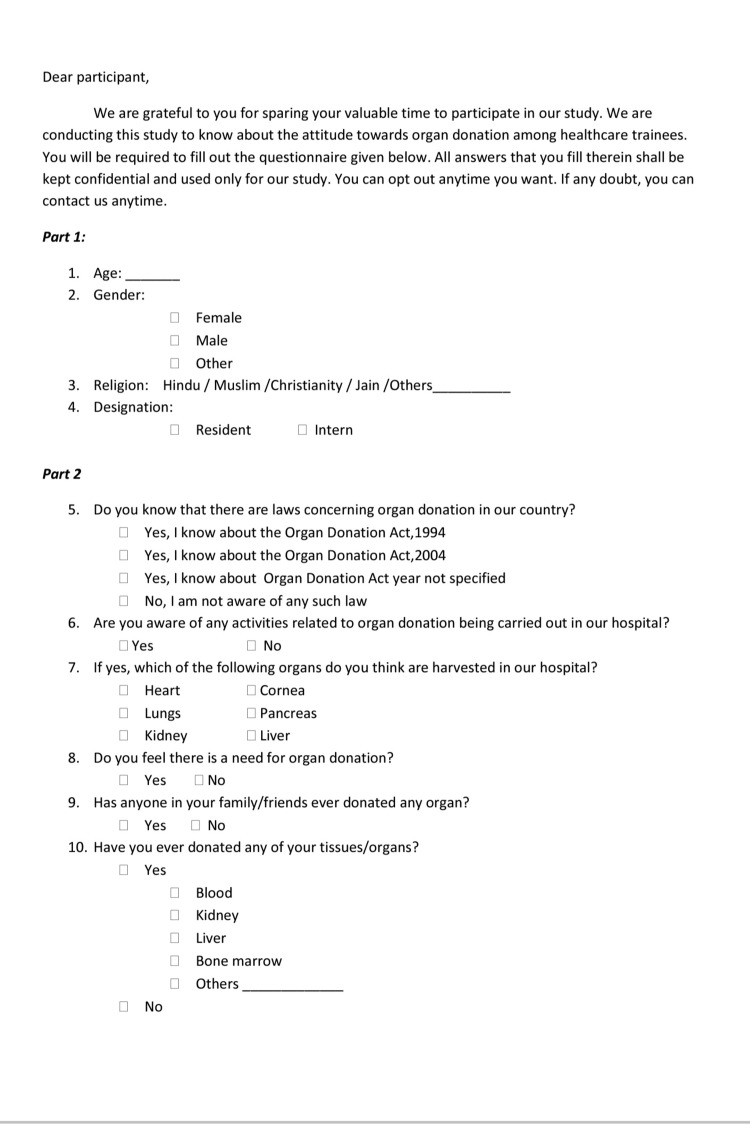
First page.

**Figure 4:**
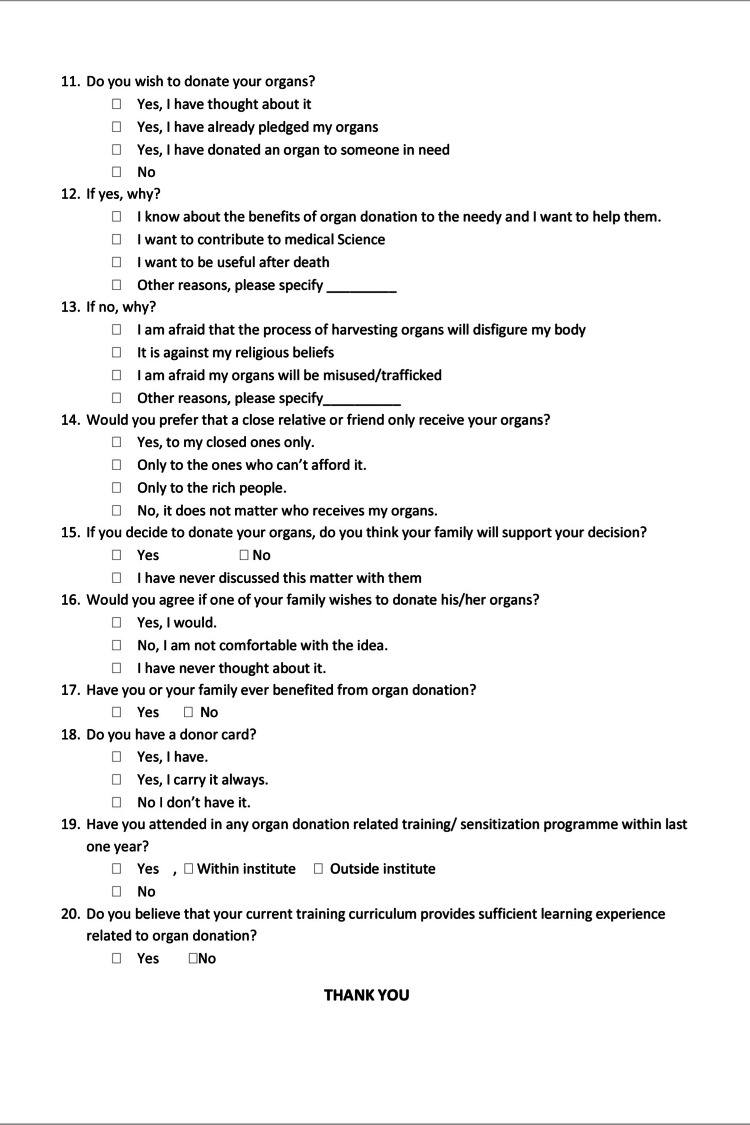
Second page.

## References

[1] (2023). The Transplantation of Human Organs Act. https://main.mohfw.gov.in/sites/default/files/Act%201994.pdf.

[2] Rosen RD, Singh A, Burns B (2023). Trauma Organ Procurement. https://europepmc.org/article/nbk/nbk555947.

[3] (2023). Ministry of Health and Family Welfare. Government of India. Lok Sabha unstarred question no 1563. https://www.mohanfoundation.org/loksabha/loksabhaQA.asp.

[4] (2022). Deceased organ donation data in India. https://www.organindia.org/deceased-organ-donation-data/.

[5] (2023). Situation in India and data. Organ receiving and giving awareness network. https://www.organindia.org/overviews/know-organ-donation/.

[6] (2023). National Organ & Tissue Transplant Organization. Ministry of Health and Family Welfare. Government of India. https://www.notto.gov.in/organreport.htm.

[7] Nallusamy S, Shyamalapriya Shyamalapriya, Balaji Balaji, Ranjan Ranjan, Yogendran Yogendran (2018). Organ donation - current Indian scenario. J Pract Cardiovasc Sci.

[8] Bathija G, Ananthesh BG, Bant DD (2017). Study to assess knowledge and attitude towards organ donation among interns and post graduates of a medical college in Karnataka, India. Natl J Community Med.

[9] Adithyan GS, Mariappan M, Nayana KB (2017). A study on knowledge and attitude about organ donation among medical students in Kerala. Indian J Transpl.

[10] Jothula KY, Sreeharshika D (2018). Study to assess knowledge, attitude and practice regarding organ donation among interns of a medical college in Telangana, India. Int J Community Med Public Health.

[11] Bharambe VK, Arole VU, Puranam V, Kulkarni PP, Kulkarni PS (2018). Knowledge and attitude toward organ donation among health-care professionals in a rural town in India. Saudi J Kidney Dis Transpl.

[12] Kumar G, Verma R, Agrawal G, Sachdeva A (2020). Knowledge and attitude regarding organ donation among undergraduate medical students in a tertiary healthcare centre. Int J Community Med Public Health.

[13] Morgan SE, Harrison TR, Afifi WA, Long SD, Stephenson MT (2008). In their own words: the reasons why people will (not) sign an organ donor card. Health Commun.

[14] Annadurai K, Mani K, Ramasamy J (2013). A study on knowledge, attitude and practices about organ donation among college students in Chennai, Tamil Nadu-2012. Prog Health Sci.

[15] Bapat U, Kedlaya PG (2010). Organ donation, awareness, attitudes and beliefs among post graduate medical students. Saudi J Kidney Dis Transpl.

[16] Balajee K, Ramachandran N, Subitha L (2016). Awareness and attitudes toward organ donation in rural Puducherry, India. Ann Med Health Sci Res.

[17] Devi K, Leondra L, Poovitha R (2018). Knowledge, attitude and practice of organ donation in urban areas of Puducherry - a Community based study. Public Health Rev Int J Public Health Res.

[18] Agarwal S (2015). Are medical students having enough knowledge about organ donation. IOSR J Dent Med Sci.

[19] Sahana BN, Sangeeta M (2015). Knowledge, attitude and practices of medical students regarding organ donation. Int J Curr Res Rev.

[20] (2023). Health Resources and Services Administration. Organ donation statistics. https://www.organdonor.gov/learn/organ-donation-statistics.

